# Nutritional Compositions and Phytochemical Properties of the Edible Flowers from Selected Zingiberaceae Found in Thailand

**DOI:** 10.3389/fnut.2018.00003

**Published:** 2018-02-01

**Authors:** Apinya Rachkeeree, Kuttiga Kantadoung, Ratchuporn Suksathan, Ratchadawan Puangpradab, Paul Alexander Page, Sarana Rose Sommano

**Affiliations:** ^1^Queen Sirikit Botanic Garden, The Botanical Garden Organization, Chiang Mai, Thailand; ^2^Plant Bioactive Compound Laboratory, Department of Plant and Soil Sciences, Faculty of Agriculture, Chiang Mai University, Chiang Mai, Thailand

**Keywords:** antioxidants, bioactive ingredients, ethnobotany, ginger family, Zingiberaceae

## Abstract

The nutritional compositions and phytochemical properties of eight edible flowers of the ginger family (Zingiberaceae) commonly found in Thailand are reported herein. The plant genera investigated were *Zingiber* (Ginger, Phlai Dam, Krathue), *Hedychium* (two morphological filament forms), *Curcuma* (Ao), *Etlingera* (Torch ginger), *Amomum* (Chi Kuk), and *Alpinia* (Galangal), which are eaten fresh or cooked as ingredients in the preparation of many Thai dishes. The proximate compositions (moisture, ash, fiber, protein, fat, and carbohydrate contents) varied among the different genera. The plants sampled were generally low in fat content (<1%), which contributed as little as 30% of the total caloric energy. Edible plant parts contained substantially high amounts of potassium (max. 737.21 mg/100 g), calcium (max. 140.15 mg/100 g), and iron (~0.32 mg/100 g). Among the tested samples, torch ginger had the highest vitamin C content (1.05 mg/100 g), total phenolic and total flavonoid contents, as well as 2,2-diphenyl-1-picrylhydrazyl activity. On the other hand, the 2,2′-azino-bis (3-ethylbenzothiazoline-6-sulfonic acid) assay suggested that *Hedychium* species possessed the highest antioxidant activity (~5.38 mg TEAC/g extract). Our results prove that edible plants of the Zingiberaceae family found in Thailand are rich sources of potentially important nutrients.

## Introduction

Plant species of the Zingiberaceae family are widely distributed in the tropical and subtropical regions of the world (1, 2). In Asia, Zingiberaceae can be found in South and South East Asia, particularly in humid lowland habitats or at higher altitudes (3). The Zingiberaceae comprises more than 50 genera (approximately 1,400 species), of which 26 genera and 300 species are found in Thailand alone. The main genera of these species are *Zingiber* (49 spp.), *Kaempferia* (17 spp.), *Hedychium* (22 spp.), *Curcuma* (34 spp.), *Globba* (42 spp.), *Alpinia* (17 spp.), *Amomum* (16 spp.), *Etlingera* (12 spp.), and *Caulokaempferia* (14 spp.).

Almost all parts of Zingiberaceae plants are used by mankind as a source of food (spices and flavoring agents), in traditional medicine and to produce natural dyes (4). Their rhizomes are known for their medicinal, pharmacological, and nutritional properties: the rhizomes of *Curcuma longa, Boesenbergia rotunda, Alpinia galanga*, and *Zingiber officinale* are typically used to treat diarrhea, stomachache, and flatulence (5–8). The leaves of some Zingiberaceae species are also of great culinary importance, those of *Kaempferia galanga, C. longa*, and *Elettariopsis slahmong* being key ingredients in spicy savory dishes from Peninsular Malaysia (9), and those of *Alpinia zerumbet*, once dried, are used as traditional herbal tea in Japan (10). The flowers and inflorescences of most Zingiberaceae are visually attractive and thus many species are grown as ornamentals, such as *Curcuma, Globba*, and *Kaempferia* species (11). In addition, many Zingiberaceae flowers are utilized in Thailand as ingredients of local food preparations, for instance, the inflorescences of *Etlingera elatior* and the flowers of *A. galanga* are cooked in traditional Thai meat dishes (9, 12), while the flowers and inflorescences of *C. longa* are consumed as a side dish of chili paste (13). Moreover, *Hedychium coronarium* flowers are consumed as vegetables and give a distinctive aroma in scented tea, *Zingiber zerumbet* flower buds are also consumed as vegetables, used as a spice, but also as a depurative, a stimulant and to treat stomachache (14).

Phytochemicals are biological compounds produced by plants throughout primary and secondary metabolisms. Many phytochemicals convey known nutritional, biological, and pharmacological benefits (15, 16). Recently, the impact of the phytochemical properties of food on human health and their preventive actions against diseases has caught the world’s attention (17, 18). Phenolic compounds, or “phenolics,” are strong antioxidants, which when consumed in adequate amounts are able to scavenge free radicals, to break radical chain reactions, and to chelate metals in the human body (19–21), all of which are causes of human diseases. Moreover, phenolics possess anti-inflammatory activities and potentially reduce the risks of cardiovascular diseases as well as of certain cancers (22–25). Other phytochemicals, such as flavonoids, tannins, and terpenoids are also strong antioxidants that counteract reactive oxygen species (ROS) and are known to reduce the risk of diseases such as heart failure, brain dysfunction, neurodegenerative disorders, and rheumatism (26).

The phytochemical profiles of various Zingiberaceae species have been previously reported. For instance, the leaves of *A. zerumbet* contain flavonoids and phenolic acids that possess a higher inhibition level of beta-carotene oxidation and a greater radical scavenging activity than its rhizomes (10, 27). Essential oils extracted from the rhizomes of Zingiberaceae species typically contain terpenoids such as limonene, eugenol, pinene, and geraniol, all of which are of pharmaceutical importance (28). *Zingiber cassumunar* rhizomes contain monoterpenes and terpinen-4-ol, typically used in folk medicine to treat inflammations, muscular pain, wounds, as well as skin diseases (29). Chan and Omar (30) examined the phytochemical properties of many Zingiberaceae and found that among 26 species, the leaves of *Etlingera elatior*, also known as torch ginger, had the highest phenolic content and radical scavenging activity.

Flowers are also important plant parts, as they produce distinctive scents and vivid pigments to attract insects and ensure efficient pollination. Terpenoids and phenolics are bioactive compounds that play a major role in these plant–insect interactions (15) and floral tissues are therefore potential sources of phytochemically active ingredients (31). In Thailand, many flowers are consumed for medicinal and nutritional purposes (32, 33). They are also used as ingredients in food, garnishes, or as an integral part of a dish, such as in salads, soups, entrees, desserts, and in beverages (34). Song et al. (35) and Youwei et al. (36) further showed that phenolic acids, flavonoids, anthocyanin, and other phenolic compounds are often produced in floral tissues and that they are equally valuable sources of antioxidants. Thus, not only are many Zingiberaceae flowers edible but their nutritional value is now well recognized in the food and nutraceutical industries. However, although flowers of the Zingiberaceae family are commonly used as food ingredients in Thailand, the information on their nutritional composition and on their effective phytochemical properties remain scarce. Therefore, the objective of this study was to investigate the nutritional values and the antioxidant activities, total phenolic and total flavonoid contents of eight edible Zingiberaceae flowers found and locally consumed in Northern Thailand. The information we provide here is useful to nutritionists and plant biologists that seek to promote the culinary use of local wild plant species as well as to support their ecological conservation.

## Materials and Methods

### Plant Material and Sample Preparation

The flowers of 8 species of Zingiberaceae, namely, *Curcuma plicata, Alpinia galanga, Amomum maximum, Zingiber ottensii, Z. zerumbet, Z. officinale, Hedychium forrestii* (variety with a yellow filament), *H. forrestii* (variety with an orange filament), and *Etlingera elatior* were used for our experiments. Flowers or inflorescences were either sampled from the living collections of the Queen Sirikit Botanic Garden (QBG), Chiang Mai, Thailand or purchased from local markets in Northern Thailand. Their voucher specimens were taxonimically identified according to Zingiberaceae experts’ comments (Suksathan, personal communication) and literature (3). The plant specimens were then deposited at the QBG herbarium. Information with regard to their common names, the parts of the plants consumed, and the types of food prepared with them was acquired by a two-way communication interview with local people in the areas of sampling (37). Based on the interview, the flowers or inflorescence parts were collected and divided into two groups; the first group was stored at -20°C immediately after collection and kept for ascorbic acid content analysis. For the second group, the plant tissues were dried in an air force oven at 45°C for 48 h and then ground to a fine powder by using an electrical grinder (Model DMF—6A, Japan) at high speed. This group of samples was used for determination of nutritional and phytochemical compositions. The moisture content of fresh samples was also determined by drying 10 g of the plant tissue at 105°C until a constant weight was reached (38).

### Proximate and Mineral Compositions

Proximate analyses were performed according to the Association of Official Analytical Chemists (38) methods for total protein, total fat, total dietary fiber, and ash content. Total carbohydrate contents were calculated using the following equation:

$$\text{Carbohydrate content}\left(\%\right)=100-\left(\begin{array}{l} \%\text{ moisture content}\\ +\%\text{ total protein}\\ +\%\text{ ash content}\\ +\%\text{ total fat content} \end{array}\right).$$

Macroelements such as sodium (Na), potassium (K), and calcium (Ca) and one microelement, iron (Fe), were estimated according to Xiao et al. (39), by which dried powder samples (1.0 g) are first incinerated at 550°C for 16 h. Ashes were then dissolved in nitric acid and passed through an ash-free, acid-washed filter paper (Albet No. 242, 9 cm diameter). Macro- and microelements were determined with an Atomic Absorption Spectrophotometer. Standards of mineral elements for flame atomic absorption spectrophotometry were obtained from Panreac (Panreac Quí-mica SA, Barcelona, Spain).

The total energy of one serving of sample (100 g fresh weight) was calculated according to the following equation (40):

$$\begin{array}{l} \text{Total energy}=(\text{energy content of }1\text{ g protein}\times \text{g protein of sample})\\ \;+\;(\text{energy content of 1 g fat}\times \text{g fat of sample})\\ \;+\left(\begin{array}{l} \text{energy content of 1 g carbohydrate}\\ \times \text{g carbohydrate of sample} \end{array}\right), \end{array}$$

where the energy content of 1 g protein = 4 kcal, energy content of 1 g fat = 9 kcal, and the energy content of 1 g carbohydrate = 4 kcal.

### Analysis of Ascorbic Acid

HPLC analysis of ascorbic acid was used following a method modified from Asami et al. (41). Small pieces of frozen samples (1–5 g) were homogenized with 20 mL of 4.5% metaphosphoric acid (HO_3_P) in ultra turrax (IKA Laroratechnik, Malaysia) during 4 min. The extract was centrifuged at 16,096 × *g* for 15 min at 4°C, and the supernatant was filtered using filter paper (Whatman no. 1). Ascorbic acid content was then quantified by Shimadzu’s HPLC system on reversed-phase C18 column (150 mm × 4.6 mm diameters, Waters Corporation, USA) under the following conditions: injected volume 20 µL; oven temperature 40°C; solvent potassium phosphate (10 mM); flow rate 1.5 mL/min, and the detection was performed with an ultraviolet wavelength of 242 nm. Total ascorbic content was calculated from calibration curves by comparison with external standard (ascorbic acid, Sigma-Aldrich, MO, USA) (42, 43).

### Phytochemical Analyses

#### Sample Extraction

A dried powder (0.5 g) was extracted with 1.0 mL of 95% (v/v) methanol. The mixture was vortexed for 1 min, and the resulting homogenate was centrifuged at 14,000 × *g* for 10 min at 4°C. The supernatant was then collected and used for further phytochemical analyses (44).

#### Total Phenolic Content (TPC)

Total phenolic content of the samples was calculated following a modified procedure of Ao et al. (45). The methanol extract of each sample (20 µL) was transferred into a 96-well plate, and 100 µL of 10% of Folin–Ciocalteu’s reagent (v/v) was added. After 1 min, 80 µL of 7.5% of sodium carbonate (Na_2_CO_3_) solution (w/v) was added to the mixture, which was then incubated for 30 min in the dark at room temperature (25°C). Finally, the absorbance of the solution was measured at 765 nm by using a microplate spectrophotometer (EZ Read 2000, England), and the TPC was reported as milligrams of gallic acid equivalents per gram of extracted sample (mg GAE/g extract). Gallic acid was obtained from Sigma-Aldrich (Hong Kong, China).

#### Determination of Total Flavonoid Content

Total flavonoid content was assessed according to the method of Hajiaghaalipour et al. (46) with slight modifications. Briefly, 100 µL of the dried plant extract was mixed with 10 µL of 5% of sodium nitrite (NaNO_2_) solution in a 96-well plate. After 5 min incubation in the dark, 10 µL of 10% of aluminum nitrate [Al(NO_3_)_3_] solution was added, and the mixture was further incubated for 5 min in the dark at room temperature. Finally, 100 µL of 1 M sodium hydroxide (NaOH) and 30 µL of distilled water were added to the mixture and shaken. The absorbance was then measured at 510 nm by using a microplate spectrophotometer (EZ Read 2000, England). The total flavonoid contents were expressed as milligrams of rutin equivalents per gram of extracted sample (mg RE/g extract). Rutin was obtained from Sigma-Aldrich (Hong Kong, China).

#### Determination of 2,2-Diphenyl-1-Picrylhydrazyl (DPPH) Radical Scavenging Activity

2,2-Diphenyl-1-picrylhydrazyl radical scavenging activity was determined according to Yen and Hsieh (47) with slight methodological modifications. The methanol extract of each sample (67 μL/well) was transferred to a 96-well plate, and 133 µL methanolic solution of DPPH radical was added. The mixture was then incubated for 30 min in the dark and at room temperature, after which the absorbance was measured at 517 nm with a microplate spectrophotometer (EZ Read 2000, England). Inhibition (%) was calculated using the following equation:

$$\text{DPPH scavenging activity}(\%)=\left[\begin{array}{l} 1-(\;\text{absorbance of sample}\\ \text{ / absorbance of control}) \end{array}\right]\times 100,$$

where the control sample was composed of 67 µL pure methanol and 133 µL DPPH methanolic solution. Finally, the antioxidant activity of each sample of extract was expressed as the half maximal inhibitory concentration (IC_50_) value (mg/mL).

#### Determination of 2,2′-Azino-Bis (3-Ethylbenzothiazoline-6-Sulfonic Acid) (ABTS) Radical Scavenging Activity

The radical scavenging measurement of ABTS was performed according to Re et al. (48) and Chang et al. (49), with slight modifications. Initially, ABTS was dissolved in deionized water to the concentration of 7 mM, and potassium persulfate (K_2_S_2_O_8_) was added up to the concentration of 2.45 mM. The mixture was incubated at room temperature overnight (16–18 h) and in the dark before use. The stock solution of ABTS was diluted with absolute ethanol to obtain an absorbance value of 0.70–0.90 at 734 nm with a microplate spectrophotometer (EZ Read 2000, England), this solution was the working ABTS solution. The methanol extract of the sample (1.9 µL) was then pipetted in to 96-well plate, and 7.5 µL of absolute ethanol and 190.6 µL of working ABTS solution were added. The mixture was finally shaken and incubated in the dark at room temperature for 5 min, with an absorbance at 734 nm with a microplate spectrophotometer (EZ Read 2000, England). The ABTS results were expressed as milligrams of Trolox equivalent antioxidant capacity per gram of extracted sample (mg TEAC/g extract). Trolox was obtained from Sigma-Aldrich (MO, USA).

### Statistical Analysis

All experiments were performed to the least in triplicate and reported as means ± SD. Differences between samples were determined by Duncan’s multiple range tests in SPSS statistical program ver. 17 (SPSS Inc., Chicago, IL, USA). A probability level of 99% was used in testing the statistical significance of all experimental data.

## Results and Discussion

### Traditional Use of Edible Flowers from the Zingiberaceae Family

**Figure 1:**
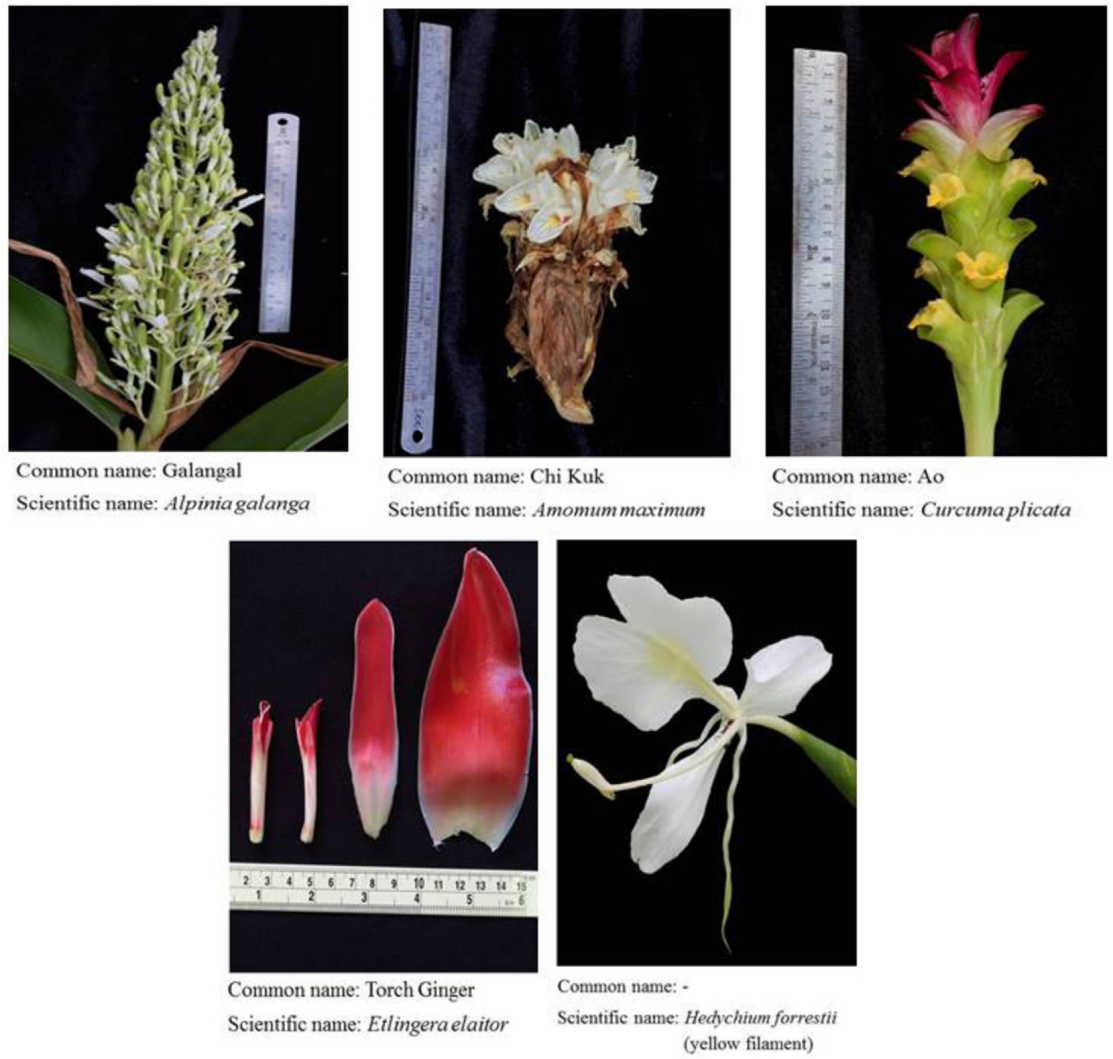
Flowers and inflorescences of *Alpinia galanga, Amomum maximum, Curcuma plicata, Etlingera elatior*, and *Hedychium forrestii* (yellow filament).

**Figure 2:**
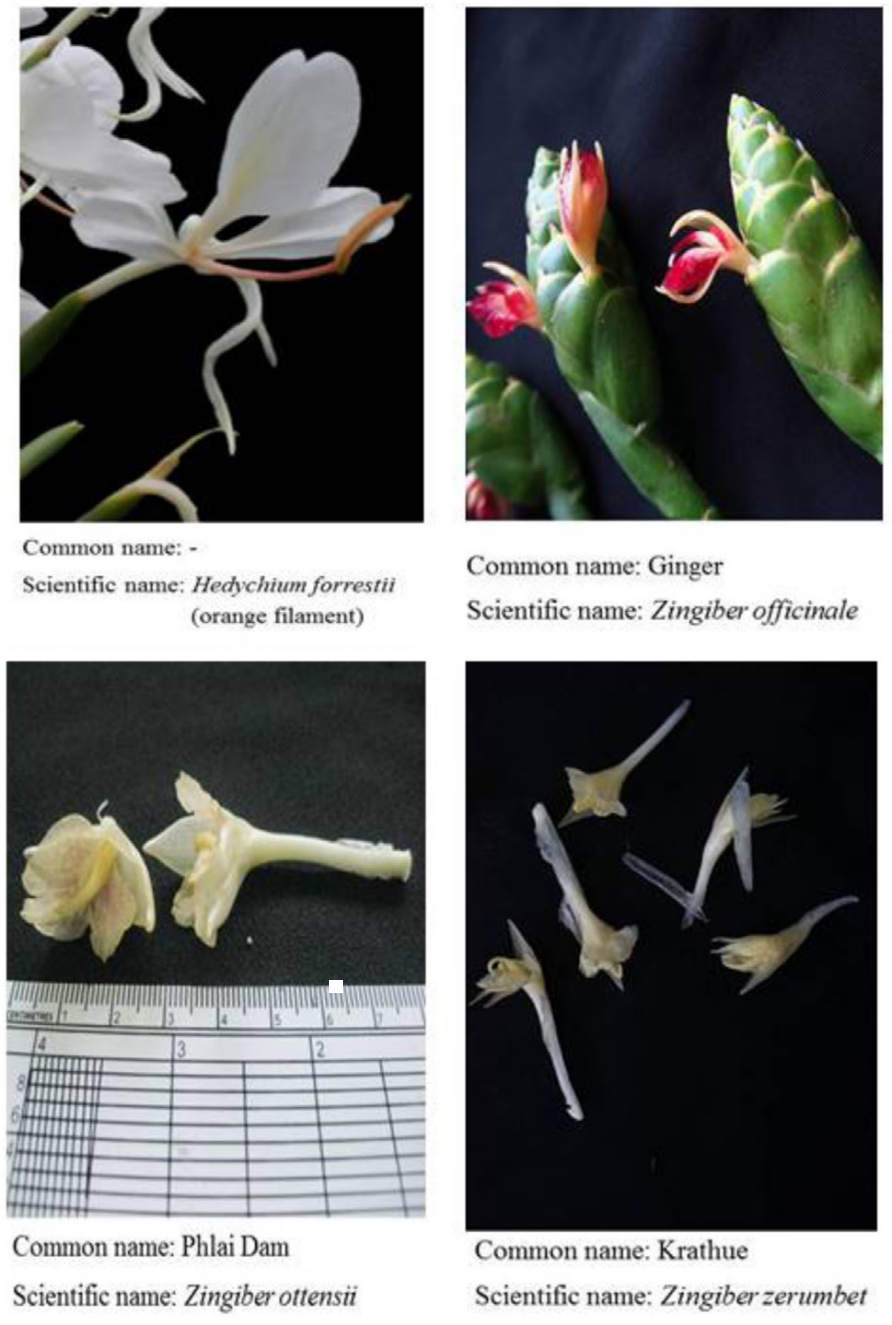
Flowers and inflorescences of *Hedychium forrestii* (orange filament), *Zingiber officinale, Zingiber ottensii*, and *Zingiber zerumbet*.

In this study, we identified and reported the common plant names, the plant parts consumed and the types of food prepared with some edible flowers of the Zingiberaceae family by using two-way communication interviews with local people in the Northern Thailand area (Tables 1**–**3; Figures 1 and 2). Our survey revealed that these edible flowers were of the *Zingiber* (Ginger, Phlai Dam, Krathue), *Hedychium* (yellow filament and orange filament), *Curcuma* (Ao), *Etlingera* (Torch ginger), *Amomum* (Chi Kuk), and *Alpinia* (Galangal) genera. The main traditional uses of the surveyed Zingiberaceae plants were for medicinal purposes, such as the use of rhizomes to treat stomach ailment and fever, and as an anti-inflammatory agent. The flowers and rhizome of *A. galanga*, fruits and seeds of *A. maximum*, the rhizome of *H. forrestii*, the inflorescence of *Z. zerumbet*, and all parts of *Z. officinale* were commonly used to treat stomach pains. This ethnobotanical knowledge confirms previous findings, by which the rhizomes of *A. galanga* and *Z. officinale* are used to treat diarrhea, stomachache, and flatulence (5). To treat fever, local Northern Thai people boil fresh inflorescences or other plant parts of *Z. zerumbet* and *Z. officinale* in water that they drink immediately after the beverage has cooled down. Other medicinal purposes gathered from our communication interviews included the use of *C. plicata* rhizome to cure constipation and the use of all *Z. officinale* plant parts to treat vomiting, diabetes, sore throats, and headaches. Moreover, the flowers of some Zingiberaceae species were used in food and beverages, such as the flowers and leaves of *E. elatior*, as previously described in Larsen et al. (9). Similarly, our survey showed that all plant parts of the common ginger, *Z. officinale*, are used as ingredients in local food and dishes. As found in our own study, Larsen et al. (9) had also reported that *Z. officinale* rhizomes are often eaten raw or cooked as vegetables and that they are sometimes added to local food dishes as spices and condiments.

**Table 1:** Scientific name, common name, and plant parts used of some Zingiberaceae family.

Scientific name	Common name	Plant part used					
		Flower	Inflorescence	Fruit	Leaf	Stem	Rhizome
*Alpinia galanga*	Galangal, Kah	✓					✓
*Amomum maximum*	Chi Kuk	✓		✓			✓
*Curcuma plicata*	Ao	✓					
*Etlingera elatior*	Torch ginger	✓			✓		
*Hedychium forrestii* (yellow filament)	Sa lay dtay, Chayheun				✓		✓
*H. forrestii* (orange filament)	Sa lay dtay, Chayheun				✓		✓
*Zingiber officinale*	Ginger, King	✓	✓		✓	✓	✓
*Z. ottensii*	Phlai Dam	✓	✓				✓
*Z. zerumbet*	Krathue	✓	✓		✓	✓	✓

**Table 2:** Scientific name and traditional use of eight Zingiberaceae plant species found in Thailand.

Scientific name	Traditional use of flowers	Traditional use of other plant parts
*Alpinia galanga*	Treatment of skin infection, vomiting, and diarrhea (50)	Treatment of stomach ailment (5–8, 50)

*Amomum maximum*		Treatment of cough, cold, vomiting, nausea, and indigestion
		Fruits and seeds: treatment of stomach ailment (51)

*Curcuma plicata*	Treatment of flatulency (13)	Treatment of stomach ailment (13, 14)

*Etlingera elatior*		Leaves: treatment for cleaning wounds and remove body odor for woman after giving birth (9, 52, 53)

*Hedychium forrestii* (yellow filament)		Treatment of stomach ailment
		Leaves: treatment for indigestion, relieve stiff, and sore joints (14, 28, 54)

*H. forrestii* (orange filament)		Treatment of stomach ailment
		Leaves: treatment for indigestion, relieve stiff, and sore joints (14, 28, 54)

*Zingiber officinale*		Rhizome: treatment of vomiting, fever, dry mouth, sore throat, headaches, stomach ailment, and diabetes (3, 5–10, 14)

*Z. ottensii*		Stem: treatment of postpartum care
		Rhizome: treatment of lumbago and convulsions (14)

*Z. zerumbet*	Treatment of stomachache (14)	Rhizome: treatment of sore throat, fever, and gastrointestinal (6, 14)

**Table 3:** Scientific name and food preparation of the flowers from eight species of the Zingiberaceae family found in Thailand.

Scientific name	Method of preparation				
	Fresh	Curry	Blanch	Chili paste	Boiled beverage
*Alpinia galanga*	✓		✓		✓
*Amomum maximum*	✓	✓	✓		
*Curcuma plicata*		✓			
*Etlingera elatior*	✓	✓			✓
*Hedychium forrestii* (yellow filament)					✓
*H. forrestii* (orange filament)					✓
*Zingiber officinale*		✓	✓	✓	✓
*Z. ottensii*		✓	✓		
*Z. zerumbet*	✓		✓		

### Proximate Composition and Total Energy

The proximate compositions of the edible flowers from the ginger family are presented in Table 4, with high fluctuations found between genera. Indeed, moisture contents varied between 89.06 and 95.12 g/100 g, ash content between 0.65 and 1.66 g/100 g, fat content ranged from 0.07 to 0.85 g/100 g, carbohydrates from 1.84 to 5.48 g/100 g, fibers from 0.58 to 3.58 g/100 g, and protein contents varied from 0.06 to 2.38 g/100 g.

**Table 4:** Nutritional compositions of edible flowers from eight species of the Zingiberaceae family found in Thailand.

Parameters	AG	AM	CP	EE	HF(Y)	HF(O)	ZO	ZOT	ZZ
Moisture content (%)	89.06 ± 0.72^f^	94.38 ± 0.87^b^	93.60 ± 0.91^c^	95.12 ± 0.35^a^	92.88 ± 0.67^d^	92.84 ± 0.78^d^	90.71 ± 0.41^e^	90.46 ± 0.28^e^	93.48 ± 0.43^c^
Ash (%)	1.46 ± 0.03^b^	0.91 ± 0.29^d,e^	1.21 ± 0.09^c^	0.65 ± 0.05^f^	0.86 ± 0.02^e^	0.86 ± 0.03^e^	1.66 ± 0.04^a^	1.42 ± 0.01^b^	0.94 ± 0.02^d^
Fat (%)	0.85 ± 0.09^a^	0.61 ± 0.12^b^	0.49 ± 0.10^c,d^	0.37 ± 0.02^d^	0.69 ± 0.11^b^	0.63 ± 0.07^b^	0.46 ± 0.02^c,d^	0.63 ± 0.01^b^	0.07 ± 0.00^e^
Carbohydrate (%)	3.64 ± 0.22^c,d^	1.84 ± 0.13^g^	3.17 ± 0.30^d^	2.46 ± 0.12^e,f^	2.15 ± 0.09^f^	4.07 ± 0.24^b^	5.48 ± 0.31^a^	3.82 ± 0.28^c,d^	3.18 ± 0.25^d^
Fiber (%)	3.58 ± 0.54^a^	0.92 ± 0.07^e^	1.47 ± 0.33^c^	0.96 ± 0.06^e^	1.04 ± 0.00^d,e^	0.69 ± 0.01^f^	0.58 ± 0.02^f^	1.97 ± 0.17^b^	1.86 ± 0.11^b^
Protein (%)	1.41 ± 0.04^c^	1.34 ± 0.15^c^	0.06 ± 0.02^g^	0.44 ± 0.00^f^	2.38 ± 0.23^a^	0.91 ± 0.27^e^	1.11 ± 0.08^d,e^	1.70 ± 0.13^b^	0.47 ± 0.07^f^
Total energy of one serving of sample (kcal/100 g fresh weight)	27 ± 0.79^b^	18 ± 0.71^d^	17 ± 0.65^d^	14 ± 0.47^f^	24 ± 1.32^c^	25 ± 0.09^c^	30 ± 0.24^a^	27 ± 0.97^b^	15 ± 1.39^e^
Calories from fat	7.65 ± 0.50^a^	5.49 ± 0.26^c^	4.41 ± 0.06^d^	3.33 ± 0.10^f^	6.21 ± 0.18^b^	5.67 ± 0.42^c^	4.14 ± 0.31^e^	5.67 ± 0.12^c^	0.63 ± 0.05^g^

*Values are mean of the triplicates ± SE*.

*AG, Alpinia galanga; AM, Amomum maximum; CP, Curcuma plicata; EE, Etlingera elatior; HF(Y), Hedychium forrestii (yellow filament); HF(O), H. forrestii (orange filament); ZO, Zingiber officinale; ZOT, Z. ottensii; ZZ, Z. zerumbet*.

*^a–g^Mean values with different superscript letter within the same column of each parameters are significantly different at P < 0.05*.

The moisture content of common leafy vegetables typically ranges between 60 and 90% (55–57). In our study, the floral tissues of *E. elatior* exhibited the highest moisture content (95.12 g/100 g), followed by *A. maximum* 94.38 g/100 g, *C. plicata* 93.60 g/100 g, and *Z. zerumbet* 93.48 g/100 g. *A. galanga* had the highest fat content (0.85 g/100 g), followed by *H. forrestii* (yellow filament), 0.69 g/100 g, *H. forrestii* (orange filament), and *Z. ottensii*, 0.63 g/100 g. The higher the moisture content, the more likely plant products become perishable (58). Ash contents of these edible flower plant species were quite low compared with other green vegetables (56, 57). The content of ash in food such as fruits and vegetables often indicates the levels of inorganic compounds, macro- and essential elements, and other mineral contents (59). The edible flowers that we tested were all low in fat content (<1%), which contributed to less than 30% of the total caloric energy (Table 4), falling within the recommended daily intake range (60, 61). Thus, the consumption of these edible flowers could be adequately recommended for adults suffering of obesity. Moreover, carbohydrate contents of these flowers were quite low (<5%) when compared with other indigenous plants consumed as vegetables in Thailand (62). Flowers of the ginger family are also a potentially high source of dietary fibers (~0.58–3.58 g/100 g) when compared with green vegetables (56). The floral tissues of *A. galanga* showed the highest dietary fiber content (3.58 g/100 g). High fiber contents are beneficial to human health as they minimize the risks of diseases and illnesses by decreasing cholesterol levels. They also reduce the risks of heart diseases and of constipation (52, 63). Protein content was the highest in *H. forrestii, Z. ottensii, A. galanga*, and *A. maximum* (~1–3%). These values are comparable to the amount of protein found in African dark green leaves consumed as vegetables (~4%) (57).

The total energy content of 100 g of fresh edible flower samples ranged from 14 to 30 kcal, among which the ginger flower *Z. officinale* was the highest. In previous studies, the total energy content of torch ginger (*E. elatior*) inflorescences, a plant of the same family, reached 315 kcal/100 g from a dried basis (52), while *Z. officinale* rhizomes produced 48 kcal/100 g from a fresh weight basis (64). These discrepancies could result from differential nutritional conditions of the tested plants, which requirements depend greatly on soil properties for growth (soil depth, texture, structure, pore space, water, and air). Roy et al. (65) reported that topsoil with a high mineral and decomposing organic material contents is optimal for most Zingiberaceae plant species. Such soil compositions are, however, unique to particular areas, which could explain differences in plant growth and in nutritional patterns.

### Mineral Compositions

The mineral compositions (potassium, calcium, and iron) of the studied edible flowers are presented in Table 5. Edible Zingiberaceae flowers contained relatively high amounts of all measured macroelements, potassium (194–737 mg/100 g), calcium (8–140 mg/100 g), and iron (0–0.32 mg/100 g). The highest potassium content was found in ginger (*Z. officinale*, 737 mg/100 g) followed by galangal (*A. galanga*, 589 mg/100 g) and Phlai Dam (*Z. ottensii*, 547 mg/100 g). Calcium was also the highest in Phlai Dam (*Z. ottensii* 140 mg/100 g), followed by torch ginger (*E. elatior*, 100 mg/100 g) and *Z. zerumbet* (83 mg/100 g). Macroelements are important nutrients that play essential roles in the biochemical pathways of the human body (66). Potassium (together with sodium) is an intra- and extracellular cation that regulates cell plasma volume through osmosis, affects pH balance, and controls nerve and muscle contraction (56, 67). Calcium is involved in the growth and maintenance of bones, teeth, and muscles (68). Iron has several important functions in the human body: it is essential in carrying oxygen from the lungs to the body tissues, in maintaining an operational immune system and in supporting energy production by the metabolism (69). In our phytochemical analyses, we only found iron in the floral tissues of common ginger (*Z. officinale*), in minor yet non-negligible amounts (0.32 mg/100 g).

**Table 5:** Microminerals and macrominerals of edible flowers from eight species of the Zingiberaceae family found in Thailand.

Parameters	AG	AM	CP	EE	HF(Y)	HF(O)	ZO	ZOT	ZZ
**Macroelements**									
Sodium (g/100 g)	–	–	–	–	–	–	–	–	–
Calcium (mg/100 g)	41 ± 1.42^e^	56 ± 1.61^d^	38 ± 1.34^f^	100 + 0.76^b^	13 ± 0.88^g^	8 ± 0.85^i^	41 ± 0.59^e,f^	140 ± 1.45^a^	83 ± 0.72^c^
Potassium (mg/100 g)	589 ± 0.89^b^	278 ± 0.78^f^	381 ± 1.04^d^	194 ± 0.95^h^	256 ± 1.86^g^	194 ± 1.32^h^	737 ± 1.73^a^	547 ± 0.97^c^	310 ± 1.10^e^

**Microelement**									
Iron (mg/100 g)	–	–	–	–	–	–	0.32 ± 0.01	–	–

*Values are means of the triplicates ± SE*.

*AG, Alpinia galanga; AM, Amomum maximum; CP, Curcuma plicata; EE, Etlingera elatior; HF(Y), Hedychium forrestii (yellow filament); HF(O), H. forrestii (orange filament); ZO, Zingiber officinale; ZOT, Z. ottensii; ZZ, Z. zerumbet*.

*^a–i^Mean values with different superscript letter within the samerow of each parameters are significantly different at P < 0.05*.

### Phytochemical Compositions

Vitamin C and total phenolic and total flavonoid contents of the edible Zingiberaceae flowers tested in our study are illustrated in Table 6. Vitamin C content varied from 0 to 1.05 mg/100 g of sample, of which torch ginger (*E. elatior*) showed the highest content (1.05 mg/100 g), followed by Phlai Dam (*Z. ottensii* 0.09 mg/100 g), and Ao (*C. plicata*, 0.08 mg/100 g). These values are relatively low when compared with Sesbania flowers (*Sesbania grandiflora*; 73.0 mg/100 g) (70). Vitamin C is an important growth factor as well as an essential antioxidant that protects plants during photosynthesis and human beings against oxidative stress produced by a range of pollutants and aerobic metabolism (71, 72). In addition, the benefits of vitamin C for human health are in promoting iron absorption, strengthening blood vessels, increasing antibody concentrations, decreasing cholesterol concentrations, preventing cardiovascular and connective tissue diseases, in healing wounds, and preventing gum bleeding (73).

**Table 6:** Phytochemical results of edible flowers from eight species of the Zingiberaceae family found in Thailand.

Phytochemicals	AG	AM	CP	EE	HF(Y)	HF(O)	ZO	ZOT	ZZ
Vitamin C (mg/100 g)	–	–	0.08 ± 0.00^b^	1.05 ± 0.03^a^	–	–	0.05 ± 0.00^c^	0.09 ± 0.00^b^	–
Total phenolics (mg GAE/g extracts)	0.28 ± 0.02^b^	0.28 ± 0.02^b^	0.16 ± 0.02^e^	2.29 ± 0.00^a^	0.30 ± 0.03^b^	0.31 ± 0.00^b^	0.14 ± 0.01^e^	0.20 ± 0.01^c,d^	0.23 ± 0.00^c^
Total flavonoids (mg RE/g extracts)	18.50 ± 0.02^f^	20.17 ± 1.15^e^	15.50 ± 0.20^i^	42.50 ± 2.64^a^	22.83 ± 0.61^d^	27.5 ± 0.46^c^	37.50 ± 0.50^b^	16.50 ± 0.73^h^	16.83 ± 0.58^g^
DPPH (% inhibition)	42.88 ± 1.36^c^	49.46 ± 0.53^b^	25.35 ± 0.77^g^	68.70 ± 1.26^a^	29.78 ± 1.52^f^	37.78 ± 0.32^e^	38.21 ± 0.81^d^	23.91 ± 1.95^h^	9.99 ± 1.99^i^
IC_50_ (mg/mL)^j^	4.60 ± 0.41^c^	4.38 ± 0.37^b^	8.48 ± 0.59^f^	2.86 ± 0.02^a^	7.10 ± 0.56^f^	5.85 ± 0.02^e^	4.87 ± 0.12^d^	10.77 ± 0.23^g^	24.72 ± 0.73^h^
ABTS (mg TEAC/g extract)	4.16 ± 0.22^c^	3.78 ± 0.07^d^	2.90 ± 0.27^h^	3.64 ± 0.03^f^	3.19 ± 0.05^g^	5.38 ± 0.11^a^	3.22 ± 0.21^e^	3.70 ± 0.22^e^	4.91 ± 0.04^b^

*Values are mean of the triplicates ± SE*.

*AG, Alpinia galanga; AM, Amomum maximum; CP, Curcuma plicata; EE, Etlingera elatior; HF(Y), Hedychium forrestii (yellow filament); HF(O), H. forrestii (orange filament); ZO, Zingiber officinale; ZOT, Z. ottensii; ZZ, Z. zerumbet; DPPH, 2,2-diphenyl-1-picrylhydrazyl; ABTS, 2,2′-azino-bis (3-ethylbenzthiazoline-6-sulfonic acid)*.

*^a–i^Mean values with different superscript letter within the samerow of each parameters are significantly different at P < 0.05*.

*^j^IC_50_ = the concentration of sample extract, which reduces the free radical DPPH about 50%*.

The TPC ranged from 0.14 to 2.29 mg GAE/g extract. Torch ginger flower (*E. elatior*) contained the highest levels of TPC at 2.29 mg GAE/g extract, while ginger flower had the lowest content at 0.14 mg GAE/g extract. Chan et al. (30) had previously reported the bioactive contents from the leaves of five *Etlingera* species and showed that *E. elatior* and *E. rubrostriata* had the highest TPCs at ~35 mg GAE/g, an ascorbic equivalent antioxidant capacity ranging from 35.40 to 37.50 mg AA/g and a ferric reducing power between 17 and 20 mg GAE/g. Wong et al. (50) reported that among five *Alpinia* species, the leaves of *A. zerumbet* and *Alpinia malaccensis* had the highest TPC at ~20 mg GAE/g. Moreover, Chan et al. (54), who screened the leaves of 26 ginger species for TPC, found that *Etlingera* sp. had the highest value (23.90 mg GAE/g) while *Hedychium* sp. had approximately 8.20 mg GAE/g of leaf extract. When comparing these results to our own, there seems to be a 10- to 15-fold increase in TPC between flower and leaf parts in some edible Zingiberaceae species of our study.

The amount of total flavonoid contents ranged from 16.53 to 42.50 mg RE/g extract. From our results, the highest total flavonoid content was found in torch ginger (*E. elatior*) flowers at 42.50 mg RE/g extract, followed by ginger flowers *Z. officinale*, at 37.50 mg RE/g extract and *H. forrestii* (orange filament) flowers, with 27.50 mg RE/g extract (Table 6) that is comparable to findings from previously published research (10, 52, 64). While phenolics and flavonoids are both known as strong antioxidants, phenolics are particularly able to scavenge free radicals, possess recognized anti-inflammatory activities, and are prone to reducing the risks of cardiovascular diseases (19–25). The antioxidant properties of flavonoids against free radicals and ROS however depend on their molecular structure and on the position of hydroxyl groups in their chemical structures (74). Nonetheless, flavonoids exhibit an excellent potential to reduce the risk of heart diseases, neurodegenerative disorders, and possess anticancer properties (26). In this study, we report the antioxidant DPPH radical scavenging activity as a percentage of inhibition against DPPH (% inhibition) and as the half maximal inhibitory concentration (IC_50_) value. Overall, the percentage of inhibition against DPPH ranged from 9.99 to 68.70%, and the IC_50_ value ranged from 2.86 to 24.72 mg/mL. Here again, torch ginger (*E. elatior*) flowers had the highest inhibition (%) and lowest IC_50_ values, which were 68.70% and 2.86 mg/mL, respectively, while Krathue (*Z. zerumbet*) showed the lowest inhibition percentage against DPPH, i.e., 9.99%, and its IC_50_ value was the highest, at 24.72 mg/mL most likely because the Krathue extract had low total contents of phenolic and flavonoid compounds, which primarily act as antioxidants. Our DPPH radical scavenging activity results however differ from those found in previous studies. Yan and Asmah (75) reported that flowers of torch ginger ground to powder had an inhibitive activity against DPPH at 11.40%, which is lower than our own result (68.70%), while the fresh form of torch ginger flower only achieved 1.45%. Maimulyanti and Prihadi (53) found that the IC_50_ of *E. elatior* flowers in a methanol extract had a higher antioxidant potential (IC_50_ = 21.14 µg/mL) than those of an ethyl acetate extract (IC_50_ = 68.24 µg/mL) against DPPH free radicals.

The ABTS radical scavenging activities of the edible Zingiberaceae flowers of our study ranged from 2.79 to 5.38 mg TEAC/g of extract. *H. forrestii* (orange filament) had the highest ABTS value (~5.38 mg TEAC/g extract), and *C. plicata* showed the lowest ABTS value (~2.90 mg TEAC/g extract). A previous study by Butsat and Siriamornpun (51) reported that the leaves of *Amomum chinense* C., another Zingiberaceae species, possess an extremely high antioxidant activity, at 46.27 µmol TEAC/g of dry weight for ABTS, when using methanol as the extracting solvent. The ABTS values were however not correlated with the results from the DPPH assay in our study, and this was most likely due to structural differences or to the location of hydroxylation, glycosylation, and methoxylation in the sampled extract (76).

## Conclusion

Edible flowers are historically part of traditional Thai cuisine and culture. They are often used as ingredients in local food and beverages, for medicinal or pharmaceutical purposes and also in religious rituals. This study acknowledges the importance of some flowers of the Zingiberaceae family that are commonly used in local food dishes by Northern Thai people. Our results illustrate their potential as sources of essential nutrients, for instance, the high dietary fiber content of galangal (*A. galanga*), the high macroelement (potassium and calcium) content of galangal, ginger (*Z. officinale*), and torch ginger (*E. elatior*) flowers, and the flowers of torch ginger as a source of strong antioxidants. Our findings thus provide useful information for the development of flower-based plant products, particularly in the food and nutraceutical industries, as well as ethnobotanical purposes to support their future ecological conservation.

## Ethics Statement

All data collections performed in the two-way communication interviews of this study were conducted according to the basic principles expressed in the Declaration of Helsinki. Informed verbal consent was obtained from all research participants, and any identifying information (such as participants’ names and locations) was removed during the preparation of the database, to protect subject anonymity. The data collections were, however, not submitted to an ethics review process, as none of the data collected were of medical or clinical purpose, would have a direct impact on the participants’ health, or affect the participants’ life and welfare.

## Author Contributions

The project idea was developed by SS; the experimental design was developed by SS and RS; sample collections were performed by AR, KK, RS, and RP; the laboratory experiments were run by AR, KK, and RP; the data were analyzed by AR, KK, and PP; and the manuscript was written by AR, KK, RS, RP, PP, and SS.

## Conflict of Interest Statement

The authors declare that the research was conducted in the absence of any commercial or financial relationships that could be construed as a potential conflict of interest.
